# Serum levels of bupivacaine after pre-peritoneal bolus vs. epidural bolus injection for analgesia in abdominal surgery: A safety study within a randomized controlled trial

**DOI:** 10.1371/journal.pone.0178917

**Published:** 2017-06-14

**Authors:** Timothy H. Mungroop, Ganapathy van Samkar, Bart F. Geerts, Susan van Dieren, Marc G. Besselink, Denise P. Veelo, Philipp Lirk, on behalf of the POP-UP trial group

**Affiliations:** 1 Department of Surgery, Academic Medical Center, Amsterdam, the Netherlands; 2 Department of Anesthesiology, Academic Medical Center, Amsterdam, the Netherlands; Universita degli Studi di Genova, ITALY

## Abstract

**Background:**

Continuous wound infiltration (CWI) has become increasingly popular in recent years as an alternative to epidural analgesia. As catheters are not placed until the end of surgery, more intraoperative opioid analgesics might be needed. We, therefore, added a single pre-peritoneal bolus of bupivacaine at the start of laparotomy, similar to the bolus given with epidural analgesia.

**Methods:**

This was a comparative study within a randomized controlled trial (NTR4948). Patients undergoing hepato-pancreato-biliary surgery received either a pre-peritoneal bolus of 30ml bupivacaine 0.25%, or an epidural bolus of 10ml bupivacaine 0.25% at the start of laparotomy. In a subgroup of patients, we sampled blood and determined bupivacaine serum levels 20, 40, 60 and 80 minutes after bolus injection. We assumed toxicity of bupivacaine to be >1000 ng/ml.

**Results:**

A total of 20 patients participated in this sub-study. All plasma levels measured as well as the upper limit of the predicted 99% confidence intervals per time point were well below the toxicity limit. In a mixed linear-effect model both groups did not differ statistically significant (p = 0.131). The intra-operative use of opioids was higher with CWI as compared to epidural (86 (SD 73) μg sufentanil *vs*. 50 (SD 32).

**Conclusions:**

In this exploratory study, the pre-peritoneal bolus using bupivacaine resulted in serum bupivacaine concentrations well below the commonly accepted toxic threshold. With CWI more additional analgesics are needed intraoperatively as compared to epidural analgesia, although this is compensated by a reduction in use of vasopressors with CWI.

**Trial registration:**

Netherlands Trial Register NTR4948

## Introduction

Adequate pain treatment is an important component of modern perioperative care and essential for a fast recovery. Choosing the optimal analgesic modality remains a topic of debate especially in major abdominal surgery. Epidural analgesia is considered by many to be the reference standard.[1] However, besides its excellent analgesic effect, there are some disadvantages associated with epidural analgesia. This includes the risk of epidural hematoma/abscess (incidence 1:1,000–6,000 in surgical patients),[2–4] failure rates of up to 30%,[5] and the need for preoperative placement in awake patients, which patients often seem to dislike and sometimes even refuse.[6]

Continuous wound infiltration (CWI) -with pre-peritoneal catheters- has become increasingly popular in recent years because of fewer alleged disadvantages, and offers a good alternative. A meta-analysis showed comparable pain scores with CWI as compared to epidural analgesia in abdominal surgery.[7] There is also evidence that CWI leads to decreased perioperative hypotension, reduced urinary retention[7] and a fast recovery,[8, 9] although the latter conclusion has been challenged.[10] In a recent randomized controlled trial, we showed CWI to be non-inferior regarding quality of analgesia as well as patient-reported outcomes in patients undergoing hepato-pancreato-biliary surgery.[6] Since earlier studies have shown CWI to be inferior to alternatives in the early postoperative phase (<24h)[11] we added a pre-peritoneal bolus after incision to improve intraoperative analgesia and decrease the use of substituting analgesics including opioids. An earlier study in laparoscopic hernia surgery showed a pre-emptive pre-peritoneal bolus with local anesthetic to be effective in reducing postoperative pain.[12] In another study an opioid bolus was combined with CWI, however this combination did not result in as effective early pain control compared to epidural analgesia.[13]

In the mentioned RCT[6] we had a case suggestive of local anesthetic toxicity after this bolus was given. After this needle bolus, one patient immediately showed ECG changes (arrhythmias) and became hypotensive (blood pressure suddenly dropped from 130/70 to 60/30). The noradrenaline infusion was already being given, and 10 mg of ephedrine was administered intravenously. That was followed by 200 microgram of adrenalin to restore circulation. Further measures were not needed. As this patient was already under general anesthesia and intubated, and as there was no surgical event at that time excepting the injection, we assumed a (partially) intravenous dose of local anesthetic resulting in high plasma levels. This bolus was not given according to the protocol, since it was done without aspiration and the needle was inserted several centimeters instead of 1–2 mm. Thus, this bolus was very likely given into the muscle. Since it is unclear to what plasma levels this needle-bolus leads when done correctly, our aim was to assess plasma levels after injection done according to protocol. We hypothesized that bupivacaine levels, when correctly applying this method, are below toxic levels but higher compared to epidural analgesia.

## Methods

### Participants

The TREND guidelines were followed for the reporting of this manuscript.[14] This was a prospective comparative open-label substudy in 20 of 105 patients who participated in the randomized controlled POP-UP trial (Netherlands Trial Registry number NTR4948). This substudy had a two-arm, open-label, parallel group design and was conducted in the main center of the original trial (Academic Medical Center).Inclusion of participants from the main trial for this substudy was done when it was logistically feasible to collect and process these samples. Analysis was done after all samples had been collected. Approval of the medical ethical committee (Medisch Ethische Toetsings Commissie AMC Amsterdam) was obtained (MEC2014_329). The trial protocol and rationale have been described elsewhere.[15] All patients gave both written and oral consent for study participation and additional blood samples. Eligible were adult patients undergoing subcostal or midline laparotomy for hepato-pancreato-biliary indications at the Academic Medical Center, Amsterdam. If any of the following criteria were present, patients were excluded: American Society of Anesthesiologists status of >3, chronic opioid use (>1 year), renal failure (an estimated glomerular filtration rate <40ml per min), contraindication for epidural analgesia, allergy for study medication, liver cirrhosis (Child-Pugh class C), or coagulopathies (international normalized ratio >1.5, partial thromboplastin time of >1.5x the mean of the normal range, platelets <80 x 10^9^ per L).

### Interventions

General anesthesia was induced in the operating room with 2–3 mg·kg^-1^ propofol (Fresenius Kabi, Zeist, the Netherlands).Besides, sufentanil was given for analgesia (Bipharma, Almere, the Netherlands), and for paralysis 0.6 mg·kg^-1^ rocuronium was given (Fresenius Kabi, Zeist, the Netherlands). The trachea was intubated, and the lungs were mechanically ventilated with pressure regulated volume controlled ventilation. After the induction, general anesthesia was maintained with sevoflurane (AbbVie, Hoofddorp, the Netherlands) at a minimal alveolar concentration of 1 and was supplemented by an additional bolus of sufentanil when deemed necessary. An arterial line was inserted into the left or right radial artery. A right jugular tri-lumen central line was inserted at the discretion of the anesthesiologist. A double lumen gastric tube and an urinary catheter were inserted. Cefazoline (Kefzol^™^) 1–2 gram and metronidazol (Flagyl^™^) 500 mg were given prophylactically (around 30 minutes prior to incision).

Besides sufentanil, additional analgesia was at the discretion of the anesthesiologist and was done according to local protocols. This included paracetamol, Metamizol, or esketamine (Eurocept Pharmaceuticals, Ankeveen, the Netherlands).

Fluid management was primarily done according to a stroke volume-, stroke volume variation-, or pulse pressure variation-guided, goal-directed fluid therapy protocol.[16]. Relevant parameters were obtained by means of FloTrac (Edwards Lifesciences) or trans-esophageal Doppler monitoring (EDM).

We monitored heart rate, blood pressure, arterial blood oxygen saturation and toxicity signs. The enhanced recovery program included preoperative nutritional optimization, normal oral nutrition up to 6 h and clear liquids up to 2 h before surgery, anti-thrombotic prophylaxis, normothermia and glycemic control.

### Pre-peritoneal bolus group

Patients received a single-shot bolus injection by the surgeon of 30 mL bupivacaine 0.25% at the start of the procedure after laparotomy in the pre-peritoneal space (i.e., between the peritoneum and the posterior transverse fascia). This procedure has been described before.[15] Step 1. After laparotomy, stretch the posterior transverse fascia manually or using a Kocher clamp. Step 2. Insert needle tip 1mm in the pre-peritoneal space. Step 3. Aspirate to exclude intra-vascular placement. If no blood is aspirated inject slowly, 10ml (in subcostal incision) or 15ml (in midline incision), in aliquots of 5 ml without using high pressure. Step 4. Repeat this 1 or 2 times on the designated locations. When in the correct plane, one should see the spreading of local anesthetic through the pre-peritoneal plane. This is the same plane in which the catheter tip is placed at the end of the procedure (see Appendix). This dosage was chosen because it is also given as a bolus immediately after placement of the catheters.[9] Adherence to the standard operating procedure of this bolus was checked in the operating room (by T.M.).

### Epidural bolus group

Other patients were treated with thoracic epidural analgesia. The epidural catheter was placed between the levels of T7 and T10 at the discretion of the anesthesiologist and topped up using bupivacaine 0·25% and sufentanil 1 μg/mL before incision. This was with a total of 10 ml in 2 boluses of 5 ml as is standard practice in our institution. After 30 minutes a continuous epidural pump was started at 6–10 ml/h bupivacaine 0·25%, resulting in a cumulative dosage in 80 minutes of 15–18.3 ml of bupivacaine 0·25%.

### Objective

To assess safety of bupivacaine after pre-peritoneal needle-bolus injection and compare them pragmatically with the plasma levels after standard epidural bolus.

### Outcomes

Our primary endpoint was plasma levels of bupivacaine after pre-peritoneal single shot needle-bolus or epidural bolus. Analysis was by intention-to-treat. Arterial blood samples were collected intraoperatively into heparin vials at 20, 40, 60, and 80 minutes after the pre-peritoneal bolus of bupivacaine in the pre-peritoneal bolus group and after epidural bolus in the epidural group. Plasma was separated and frozen at -80 degrees Celsius. We used the MaxSignal bupivacaine ELISA kit for immunoassay (Bio Scientific, Austin, Texas, USA) (See Appendix). Symptoms of toxicity of bupivacaine can occur from 1000–1500 ng/ml and seizures are associated with levels > 4500 ng/ml.[17, 18]

Baseline variables included: Gender, age, BMI, American Society of Anesthesiologists (ASA) physical status, creatinine. Secondary outcomes included: intraoperative noradrenaline use, fluids administered, intraoperative sufentanil and esketamine usage, and operative time.

### Sample size

This was an exploratory study. Due to the absence of literature regarding this pre-peritoneal needle-bolus, there was lack of evidence to facilitate a sample size calculation with confidence. The sample size of 20 patients (10 in each arm), which was decided beforehand, seemed reasonable for the goal of this exploratory analysis.

### Statistical analysis

Regarding the primary and secondary endpoints, we calculated based on 1000 bootstrap samples the mean and SD. For the primary endpoint we chose to display the upper limit of the 99% CI interval of the mean instead of for example the 95% CI since this seems more relevant to us because of the aim of our study. For baseline data we expressed median and IQR for continuous variables if non-Normally distributed, or mean and standard deviation (SD) when Normally distributed. The normal distribution was checked by visually inspecting the histograms. Missing data was considered missing at random. Dichotomous data were presented as numbers and percentages. For continuous variables, differences between groups were tested with Student’s *t*-test for normally distributed data. For non-normally distributed data the Mann-Whitney *U*-test was used. A linear mixed effect model was used using time with group interaction. P-value of significance was set at <0.05. Fisher’s exact test was used for proportions for all categorical data. Data was collected in, and analyzed with SPSS Version 22.0 (SPSS Inc., Chicago, IL, USA).

## Results

**Fig 1:**
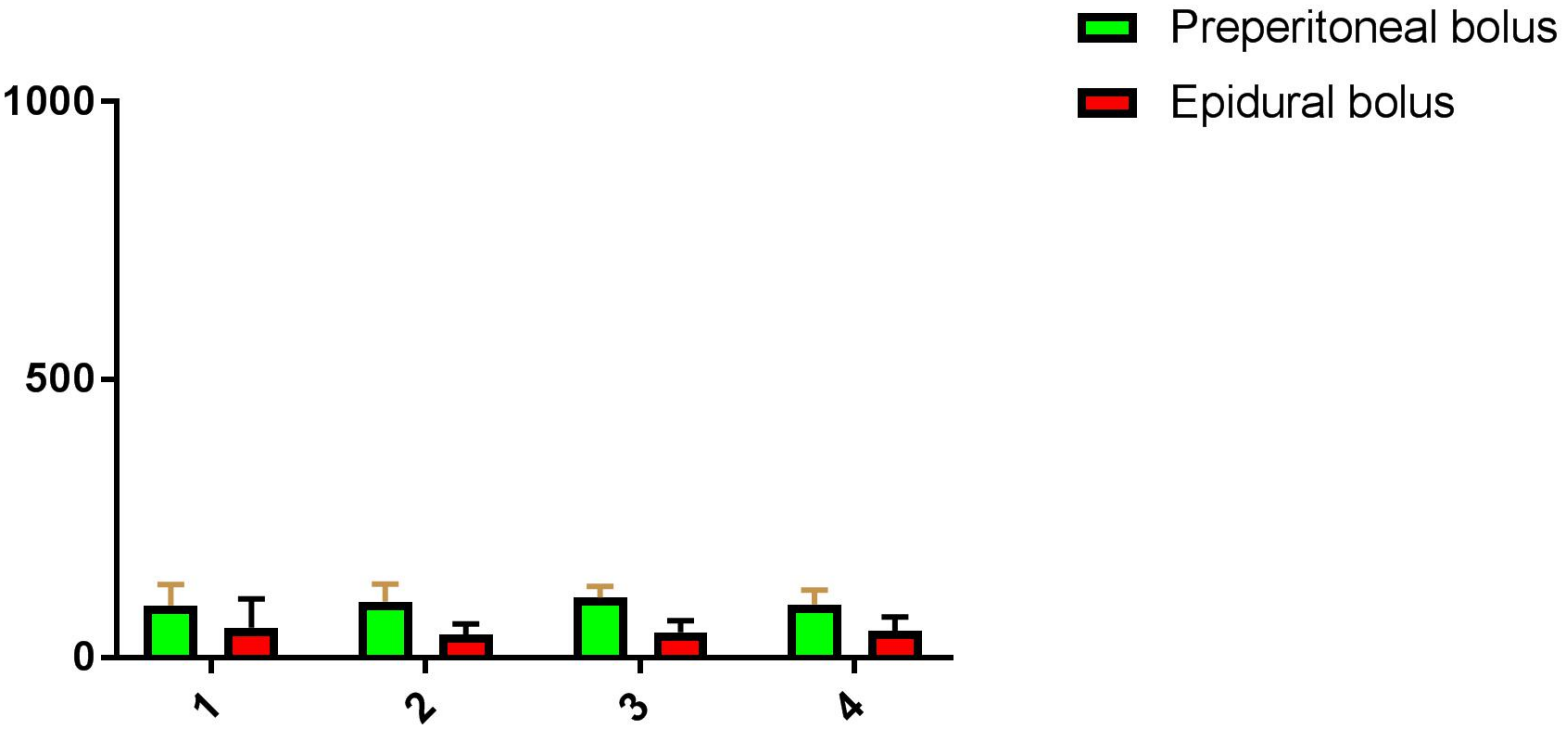
Mean plasma levels of bupivacaine per time point in ng/ml. Time point 1-2-3-4 are 20, 40, 60 and 80 minutes after bolus injection. The green bar is the mean, the error line the upper limit of the 99% confidence interval. Since toxicity symptoms can occur from 1000ng/ml we chose that as the upper limit of the Y-axis.[17, 18]

Twenty patients were included in this study between April 2015 and September 2015. All patients which were considered for these additional blood samples, agreed to participate in this substudy. Data was complete except for measurements in 2 patients in the epidural group at the 80-minute time point. There were no serious adverse events reported in these patients. For baseline data see Table 1. The time course of bupivacaine concentrations is shown in Fig 1. All plasma levels measured were well below toxic levels. The highest measurement in the pre-peritoneal bolus group was 177 ng/ml compared to 201 ng/ml in the epidural group. Also, the upper limit of the 99% confidence interval of the mean per time point never exceeded 132 ng/ml (Fig 1), which is well below toxicity. The intra-operative use of additional analgesics was higher in the pre-peritoneal bolus group (sufentanil and esketamine), (Table 2). The mixed effect model groups did not differ significantly (p = 0.131). Plasma levels of pre-peritoneal bolus injection vs epidural bolus and continuous infusion were: at 20 minutes a mean of 94 (SD 54) *vs*. 54 (54) ng/ml (p = 0.110), at 40 minutes a mean of 100 (SD 47) *vs*. 41 (21) ng/ml (p = 0.005), at 60 minutes mean of 108 (SD 28) *vs*. 45 (25) ng/ml (p<0.001) and at 80 minutes a mean of 95 (SD 35) vs 48 (28) ng/ml (p = 0.007).

**Table 1:** Baseline characteristics of the 20 participants.

	Pre-peritoneal bolus (N = 10)	Epidural bolus (N = 10)
Gender		
- Male	7 (70%)	5 (50%)
- Female	3 (30%)	5 (50%)
BMI (kg/m^2^)	24.0 [21–29.5]	24.7 [21.9–26.7]
Age (years)	60 [47–72]	75 [57–85]
ASA class		
- I	2 (20%)	1 (10%)
- II	7 (70%)	8 (80%)
- III	1 (10%)	1 (10%)
Creatinine (μmol/L)	73 [64–81]	75 [68–91]

Data are median (interquartile range) and counts (%). ASA = American Society of Anesthesiology physical status. Groups did not differ statistically significant for all baseline variables.

**Table 2:** Operative data.

	Pre-peritoneal bolus (N = 10)	Epidural bolus (N = 10)	*p*
Duration of surgery (min)	319 (SD 55)	222 (SD 41)	0.288
Norepinephrine (mg)	0.76 (SD 1.2)	1.3 (SD 1.0)	0.325
Fluids administered (ml)	3099 (SD 1507)	3285 (SD 1237)	0.890
Sufentanil (μg)	86 (SD 73)	50 (SD 32)	0.206
Esketamine (mg)	27 (SD 32)	1 (SD 3)	0.039

## Discussion

This comparative sub-study within a randomized controlled trial found that plasma levels of bupivacaine in patients receiving pre-peritoneal bolus injections are well below toxic levels. Furthermore, the intraoperative amount of analgesics used is still higher in patients with CWI compared to patients with epidural analgesia. This is indicates the pre-peritoneal bolus does not totally compensate intraoperatively for lack of epidural analgesia.

In our study, all measured levels were well below the toxic threshold on the different time points. In 51 RCTs, no cases of local anesthetic toxicity have been reported with continuous wound infiltration.[19] However, there are documented reports of toxicity; after malfunctioning of an elastomeric balloon pump,[20] and after a TAP block following partial intramuscular injection.[21]

In our POP-UP trial we experienced one serious adverse event probably linked to an accidental intravascular injection. This bolus was not given according to the protocol. Instead of inserting the needle 1-2mm, the needle was inserted 2–3 centimeters, and the bolus was given without prior aspiration. When the bolus is given in the correct plane, there is visual feedback when the local anesthetic spreads through the pre-peritoneal plane. We advise to routinely aspirate prior to injection and inject slowly in aliquots of 5 ml, without high pressures, as is common practice in regional analgesia. At the end of the operation, when the bolus is given through the pre-peritoneal catheters, there is visual and tactile feedback when the catheters curl up in the pre-peritoneal plane and the risk of intravascular injection would seem negligible.

We showed that a pre-peritoneal needle-bolus with 30ml bupivacaine 0.25% to cover the early intra-operative period results in serum bupivacaine concentrations well below the concentration commonly accepted as toxic. However, close attention needs to be paid to the execution in operating theatres. Benefits during the intraoperative period include a decrease in perioperative hypotension.[6] In our trial, this manifested as reduced use of perioperative vasopressors. No difference was found in the use of iv-fluids, which we contribute to the use of perioperative goal directed fluid therapy. Without this method, this would probably lead to infusion of more iv fluids including all associated risks.

This study has several limitations. First, multiple testing might have influenced results of our analysis. We chose to not use the Bonferroni correction, since this is not advised by some, but chose to instead report our results unadjusted.[22] Second, we only have an exploratory sample size of 20 patients. To draw definitive conclusions and declare safety a larger-scale trial is needed. However, in our opinion these results provide relevant exploratory information related to this novel addition to the technique of CWI. Besides, our results can be used in the planning of such a large-scale study. Third, the plasma levels in the epidural group are influenced by the continuous infusion of epidural bupivacaine, started 30 min after epidural bolus. However, we made the pragmatic choice to compare 2 different dosages to evaluate the plasma levels as they are with the use of both methods in daily practice. Our goal was not to compare these measurements directly, since for that purpose an equipotent dosage would be needed. Instead, we aimed to give the reader an idea to what extent these levels differ with the use of these methods as they are in daily practice. Besides, there are only measurements on the chosen time points (20, 40, 60, 80 minutes). These were chosen because of the expected epidural resorption peak at around 30 minutes, so peaks outside these time points could have been missed, but are very unlikely. A comparable study in transversus abdominis plane block showed a comparable curve, suggesting these time points were chosen correctly, without for example a very early peak.[23] However, because all measurements as well as the predicted upper limits of the 99%-CI of the mean are well below toxicity, (<180 ng/ml compared to a toxicity limit of 1000 ng/ml), we feel confident that the current intervention when correctly executed does result in relatively low levels of local anesthetic. This is the first study in which this bolus injection is evaluated, studies evaluating the pharmacokinetics and precise method of action are warranted.

## Conclusions

In this exploratory study, the pre-peritoneal bolus using bupivacaine resulted in serum bupivacaine concentrations well below the commonly accepted toxic threshold. With CWI additional analgesics are still needed intraoperatively as compared to epidural analgesia, although this is compensated by a reduction in use of vasopressors with CWI.

## Appendix

Original text of manufacturer

The MaxSignal^®^ Bupivacaine ELISA Kit uses a competitive immunoassay method to determine the amount of bupivacaine present in the blood. The MaxSignal^®^ Bupivacaine ELISA Kit uses a competitive immunoassay method to determine the amount of bupivacaine present in the blood or urine sample. Test plate wells are coated with bupivacaine. Blood or urine sample is added for analysis, along with anti-bupivacaine antibody. Bupivacaine in the sample will compete for the primary antibody, thereby preventing the antibody from binding to the drug attached to the well. After incubation, the sample is removed, the wells are washed and a secondary HRP-conjugated antibody is added. The intensity of the absorbance at 450 nm is directly proportional to the amount of Bupivacaine in the urine sample.

## Supporting information

S1 Table Dataset of this study. (XLS)

## References

[1] PROSPECT. PROSPECT Colonic Resection Subgroup. 2009.

[2] Popping DM, Zahn PK, Van Aken HK, Dasch B, Boche R, Pogatzki-Zahn EM. Effectiveness and safety of postoperative pain management: a survey of 18 925 consecutive patients between 1998 and 2006 (2nd revision): a database analysis of prospectively raised data. British journal of anaesthesia. 2008;101(6):832–40. Epub 2008/10/24. doi: 10.1093/bja/aen300 . 18945716 10.1093/bja/aen300

[3] Christie IW, McCabe S. Major complications of epidural analgesia after surgery: results of a six-year survey. Anaesthesia. 2007;62(4):335–41. Epub 2007/03/27. doi: 10.1111/j.1365-2044.2007.04992.x . 17381568 10.1111/j.1365-2044.2007.04992.x

[4] Moen V, Dahlgren N, Irestedt L. Severe neurological complications after central neuraxial blockades in Sweden 1990–1999. Anesthesiology. 2004;101(4):950–9. Epub 2004/09/28. . 15448529 10.1097/00000542-200410000-00021

[5] Hermanides J, Hollmann MW, Stevens MF, Lirk P. Failed epidural: causes and management. British journal of anaesthesia. 2012;109(2):144–54. Epub 2012/06/28. doi: 10.1093/bja/aes214 . 22735301 10.1093/bja/aes214

[6] Mungroop TH, Veelo DP, Busch OR, van Dieren S, van Gulik TM, Karsten TM, et al Continuous wound infiltration versus epidural analgesia after hepato-pancreato-biliary surgery (POP-UP): a randomised controlled, open-label, non-inferiority trial. Lancet Gastroenterol Hepatol 2016. 2016 Epub 07-07-2016. http://dx.doi.org/10.1016/S2468-1253(16)30012-7. 10.1016/S2468-1253(16)30012-7 28404067

[7] Ventham NT, Hughes M, O'Neill S, Johns N, Brady RR, Wigmore SJ. Systematic review and meta-analysis of continuous local anaesthetic wound infiltration versus epidural analgesia for postoperative pain following abdominal surgery. The British journal of surgery. 2013;100(10):1280–9. Epub 2013/11/20. . 24244968 10.1002/bjs.9204

[8] Revie EJ, McKeown DW, Wilson JA, Garden OJ, Wigmore SJ. Randomized clinical trial of local infiltration plus patient-controlled opiate analgesia vs. epidural analgesia following liver resection surgery. HPB: the official journal of the International Hepato Pancreato Biliary Association. 2012;14(9):611–8. Epub 2012/08/14. doi: 10.1111/j.1477-2574.2012.00490.x ; 22882198 10.1111/j.1477-2574.2012.00490.x PMC3461387

[9] Hughes MJ, Harrison EM, Peel NJ, Stutchfield B, McNally S, Beattie C, et al Randomized clinical trial of perioperative nerve block and continuous local anaesthetic infiltration via wound catheter versus epidural analgesia in open liver resection (LIVER 2 trial). The British journal of surgery. 2015 Epub 2015/10/09. doi: 10.1002/bjs.9949 . 26447461 10.1002/bjs.9949

[10] Jouve P, Bazin JE, Petit A, Minville V, Gerard A, Buc E, et al Epidural versus continuous preperitoneal analgesia during fast-track open colorectal surgery: a randomized controlled trial. Anesthesiology. 2013;118(3):622–30. Epub 2013/02/22. doi: 10.1097/ALN.0b013e3182800d94 . 23426208 10.1097/ALN.0b013e3182800d94

[11] Soliz JM, Gebhardt R, Feng L, Dong W, Reich M, Curley S. Comparing epidural analgesia and ON-Q infiltrating catheters for pain management after hepatic resection. Open Journal of Anesthesiology. 2013;3(1):3–7. Epub 2013/01/01. doi: 10.4236/ojanes.2013.31002 ; 25580374 10.4236/ojanes.2013.31002 PMC4286355

[12] Hon SF, Poon CM, Leong HT, Tang YC. Pre-emptive infiltration of Bupivacaine in laparoscopic total extraperitoneal hernioplasty: a randomized controlled trial. Hernia: the journal of hernias and abdominal wall surgery. 2009;13(1):53–6. Epub 2008/08/16. doi: 10.1007/s10029-008-0422-9 . 18704618 10.1007/s10029-008-0422-9

[13] Ball L, Pellerano G, Corsi L, Giudici N, Pellegrino A, Cannata D, et al Continuous epidural versus wound infusion plus single morphine bolus as postoperative analgesia in open abdominal aortic aneurysm repair: a randomized non-inferiority trial. Minerva anestesiologica. 2016;82(12):1296–305. Epub 2016/08/31. . 27575452

[14] Grendar J, Jutric Z, Leal JN, Ball CG, Bertens K, Dixon E, et al Validation of Fistula Risk Score calculator in diverse North American HPB practices. HPB: the official journal of the International Hepato Pancreato Biliary Association. 2017 Epub 2017/02/25. doi: 10.1016/j.hpb.2017.01.021 . 28233672 10.1016/j.hpb.2017.01.021

[15] Mungroop TH, Veelo DP, Busch OR, van Dieren S, van Gulik TM, Karsten TM, et al Continuous wound infiltration or epidural analgesia for pain prevention after hepato-pancreato-biliary surgery within an enhanced recovery program (POP-UP trial): study protocol for a randomized controlled trial. Trials. 2015;16(1):562 Epub 2015/12/15. doi: 10.1186/s13063-015-1075-5 . 26654448 10.1186/s13063-015-1075-5 PMC4674956

[16] Kuper M, Gold SJ, Callow C, Quraishi T, King S, Mulreany A, et al Intraoperative fluid management guided by oesophageal Doppler monitoring. BMJ (Clinical research ed). 2011;342:d3016 Epub 2011/05/26. doi: 10.1136/bmj.d3016 . 21610051 10.1136/bmj.d3016

[17] Dillane D, Finucane BT. Local anesthetic systemic toxicity. Canadian journal of anaesthesia = Journal canadien d'anesthesie. 2010;57(4):368–80. Epub 2010/02/13. doi: 10.1007/s12630-010-9275-7 . 20151342 10.1007/s12630-010-9275-7

[18] Suresh S, De Oliveira GS. Blood Bupivacaine Concentrations After Transversus Abdominis Plane Block in Neonates: A Prospective Observational Study. Anesthesia and analgesia. 2016;122(3):814–7. Epub 2015/11/19. doi: 10.1213/ANE.0000000000001088 . 26579846 10.1213/ANE.0000000000001088

[19] Liu SS, Richman JM, Thirlby RC, Wu CL. Efficacy of continuous wound catheters delivering local anesthetic for postoperative analgesia: a quantitative and qualitative systematic review of randomized controlled trials. Journal of the American College of Surgeons. 2006;203(6):914–32. Epub 2006/11/23. doi: 10.1016/j.jamcollsurg.2006.08.007 . 17116561 10.1016/j.jamcollsurg.2006.08.007

[20] Baulig W, Maurer K, Theusinger OM, Hinselmann V, Baulig B, Spahn DR, et al Continuous elastomeric pump-based ropivacaine wound instillation after open abdominal aortic surgery: how reliable is the technique? The heart surgery forum. 2011;14(1):E51–8. Epub 2011/02/25. doi: 10.1532/HSF98.20101089 . 21345776 10.1532/HSF98.20101089

[21] Weiss E, Jolly C, Dumoulin JL, Meftah RB, Blanie P, Laloe PA, et al Convulsions in 2 patients after bilateral ultrasound-guided transversus abdominis plane blocks for cesarean analgesia. Regional anesthesia and pain medicine. 2014;39(3):248–51. Epub 2014/04/01. doi: 10.1097/AAP.0000000000000088 . 24682078 10.1097/AAP.0000000000000088

[22] Perneger TV. What's wrong with Bonferroni adjustments. BMJ (Clinical research ed). 1998;316(7139):1236–8. Epub 1998/05/16. ; 9553006 10.1136/bmj.316.7139.1236 PMC1112991

[23] Yasumura R, Kobayashi Y, Ochiai R. A comparison of plasma levobupivacaine concentrations following transversus abdominis plane block and rectus sheath block. Anaesthesia. 2016;71(5):544–9. Epub 2016/03/08. doi: 10.1111/anae.13414 . 26945692 10.1111/anae.13414

